# Elimination through the Stomach

**Published:** 1896-03

**Authors:** 


### Elimination Through the Stomach.

Experiments have been made recently on healthy dogs to determine what substances find their way into the stomach when introduced into the system by rectal or hypodermic injection. The alkaloids discovered in the vomit withdrawn mechanically from the stomach were morphin, brucin, veratrin, caffein and quinin. There was absence of atropin and apomorphin. Other substances found were salicylic acid and antipyrin. Phenic acid was not eliminated by the stomach. Still other substances noticed were chloroform, chloral hydrate, methyl alcohol ethyl alcohol and acetone. The methyl alcohol was administered in a rectal injection. It was mostly eliminated in the urine the day after the injection. These substances found their way into the stomach through the blood in some cases, but Grutzner's recent experiments show that there is an antiperistaltic action of the bowels to which may be due the presence in the stomach of some of the substances introduced into the rectum.—*Schmidt's Jahrbch.*, 1895, No. 11; reviewed in *Les Nouv. Remedes*, January 8th, 1896.

